# The Saskatchewan Movement Disorders Program: Commitment Pays Off

**DOI:** 10.1017/cjn.2015.9

**Published:** 2015-03

**Authors:** Anthony E. Lang, A. Jon Stoessl

**Affiliations:** University of Toronto; University of British Columbia

**Keywords:** Movement disorders, Parkinson disease

Dr. Ali Rajput and the program he personally established at the University of Saskatoon, starting in the late 1960s, have contributed immeasurably to our current knowledge of Parkinson’s disease (PD) and related movement disorders. One could simply state that Ali Rajput accepted a one year contract at the University of Saskatoon in July of 1967 and “the rest is history”. In this issue of the Canadian Journal of Neurological Sciences we have the great pleasure of hearing that history told in the first person. One is immediately struck by the remarkable impact of a series of simple, unexpected early events, such as the death of his first chairman while Dr. Rajput was waiting to give formal notice of resignation and departure; his subsequent betrothal to Karla, his wife now of more than 40 years; the reports of Cotzias and Barbeau on the effects of high doses of levodopa for PD; and the demand of Health Canada that Rajput conduct research in a special clinic to be able to obtain this treatment for his patients. Others placed in these circumstances may have responded effectively and subsequently developed a successful career in subspecialty neurological care and possibly research. However, the reader cannot proceed through this history without being impressed that this is not merely the story of a brighter than average neurologist who happened to be in the right place at the right time. The vital importance of the “right person” is repeatedly evident as each event or landmark in the development of the Saskatchewan Movement Disorders Program is described. This history regularly highlights the important attributes that account for the Program’s success including dedication to patients, extreme hard work, perseverance despite major setbacks, inquisitiveness and enthusiasm to pursue a research agenda and a dogged determination to create a unique and unparalleled resource that would not simply serve and enhance the reputation of the local program, but that could be utilized by colleagues and other researchers throughout the world.

In an age when many clinicians despair that they are unable to contribute meaningful research because they are not trained in the powerful but challenging and rapidly changing techniques of modern neuroscience, the work described by the Rajputs is a testament to what can be accomplished with persistence and commitment to careful longitudinal clinical assessment, a focus on addressing clinically relevant questions, and the ability to work with others. It is quite remarkable that some 25-30% of the patients followed in their clinic who died came to autopsy (an even higher proportion of those with Parkinson’s). The brain bank they established, with the support and collaboration of Oleh Hornykiewicz, himself a true hero of the Parkinson world, is the envy of investigators worldwide, not only because of the large number of carefully stewarded samples, but also because of the detailed clinical correlations made possible through their efforts. This has attracted the attention of basic neuroscientists interested in the mechanisms underlying complications of treatment in PD and led to highly fruitful collaborations with Paul Bedard, Therese di Paolo, Frederic Calon and others at Laval University. This determination has also led to a number of the very few pathological insights into mechanisms underlying essential tremor. Importantly, the contributions of the Rajputs are also a testament to the benefits of a publicly funded health care system. For many years, a high proportion of PD patients in the province of Saskatchewan were assessed and followed (even after death) by the senior author. This would likely not be possible in a free-market system where neurologists are competing for patients.

In addition to the important qualities listed above that contributed to the success of the program, three others are inadequately depicted in this type of autobiographical piece, but need to be emphasized by those of us who are fortunate to know Ali Rajput personally. These are his profound humility, his wonderful good nature and his remarkable professional generosity. How much can be accomplished by the last of these is an important lesson for faculty and trainees alike. Ali founded the Canadian Movement Disorders Group because he felt the Canadian community should be brought together and would accomplish more if it spoke (and negotiated with industry) with a single voice. He has consistently promoted the careers of younger colleagues by inviting them to serve as visiting professors, ensuring that they are included in activities of the professional community, and nominating them for prestigious awards. We have both been fortunate in our careers to have been mentored and supported by senior colleagues, including Ali, who had nothing to gain by our success other than the knowledge that they had somehow contributed. Ali Rajput must surely stand out as an exemplar of such generous behavior.

A barrage of medical literature crosses the busy neurologist’s desk on a weekly basis and the neurology resident needs to further assimilate a huge amount of basic neurological knowledge in addition to keeping up with new developments. Given these challenges it would be very easy to scan the title of this historical review and move on, especially if one has little interest in the field of movement disorders. However, those who take the time to read this review of the history and accomplishments of Dr. Ali Rajput and his son Alex in establishing and building the Saskatchewan Movement Disorders Program will be greatly rewarded by this unique and important component of the Canadian neurological sciences landscape. We strongly recommend that neurology education program directors encourage their residents to read the paper, particularly those who are considering an academic career; reviewing Dr. Rajput’s story should stimulate and reassure young trainees that they too can succeed in clinical research but it will remind them that success requires far more than basic knowledge, a good research question and grantsmanship.

